# Confirmatory factor analysis of the thyroid-related quality of life questionnaire ThyPRO

**DOI:** 10.1186/s12955-014-0126-z

**Published:** 2014-09-10

**Authors:** Torquil Watt, Mogens Groenvold, Nina Deng, Barbara Gandek, Ulla Feldt-Rasmussen, Åse Krogh Rasmussen, Laszlo Hegedüs, Steen Joop Bonnema, Jakob Bue Bjorner

**Affiliations:** Department of Endocrinology, Copenhagen University Hospital Rigshospitalet, Blegdamsvej 9, 2100 Copenhagen Ø, Denmark; Institute of Public Health, University of Copenhagen, Øster Farimagsgade 5, 1014 Copenhagen K, Denmark; Department of Palliative Medicine, Bispebjerg Hospital, Bispebjerg Bakke 3, 2400 Copenhagen N, Denmark; Department of Quantitative Health Sciences, University of Massachusetts Medical School, 368 Plantation Street, 01605 Worcester, MA USA; Department of Endocrinology and Metabolism, Odense University Hospital, Kløvervænget 6, 5000 Odense M, Denmark; National Research Centre for the Working Environment, Lersø Parkallé 105, 2100 Copenhagen, Denmark; OptumInsight, 24 Albion Rd, 02865 Lincoln, RI USA

**Keywords:** Patient-reported outcomes, Unidimensionality, Quality of life, Scale validation, Thyroid disease

## Abstract

**Background and aim:**

Thyroid diseases are prevalent and chronic. With treatment, quality of life is restored in most, but not all patients. Construct validity of the thyroid-related quality of life questionnaire, ThyPRO, has been established by multi-trait scaling, but not evaluated with more elaborate methods. The purpose of the present study was to evaluate dimensionality of the ThyPRO scales and to attempt to understand possible item misfit through structural equation modeling for categorical data.

**Methods:**

The current 85-item version of ThyPRO consists of 13 scales, covering domains of physical (4 scales) and mental (2 scales) symptoms, function and well-being (3 scales) and participation/social function (4 scales). The data were collected from a cross-sectional sample of 907 thyroid patients. One-factor confirmatory models were fitted to each scale, and evaluated by model fit statistics (comparative fit index >0.95, root mean square error of approximation <0.08), magnitude of factor loadings, model residual correlations and modification indices (MI). Indications of multi-dimensionality were tested in bi-factor models. Possible item misfit was evaluated in a combined, investigational model.

**Results:**

Each ThyPRO scale was adequately represented by a unidimensional model after minor revisions. Eleven items were identified in the unidimensional models as potentially misfitting and were investigated further by multidimensional modeling.

**Conclusion:**

Elaborate psychometric modeling supported the construct validity of the ThyPRO. However, 11 potentially misfitting items and 18 items with local dependence to other items are candidates for removal in future item reduction processes.

## Introduction

Thyroid diseases are diseases related to the thyroid gland, which is an endocrine, i.e. hormone producing, gland located in the front of the neck. Thyroid diseases are prevalent, affecting approximately 15% of individuals of all ages, with a 4 to 1 women/men ratio [1,2]. The main disease groups comprise *non-toxic goiter* (enlargement of the gland), *hyperthyroidism* (either as *toxic nodular goiter* or *Graves’ disease* -with or without *Graves’ orbitopathy* (GO, inflammation and protrusion of the eyes)) - and *autoimmune hypothyroidism*. The symptomatology is often diffuse, sharing features with many other diseases (fatigue, palpitations, dry skin, depression, uneasiness, etc.) as well as with the non-pathological fluctuations of well-being and function in life. Therefore, thyroid diseases may go un-diagnosed for many years in some patients and at the time of diagnosis, most patients have reduced quality of life [3,4]. The diseases are chronic, but relevant treatment is available. In general though, there is a lag in treatment effect for thyroid diseases of up to several months and population-based studies document excess morbidity and mortality, also when adequately treated [5,6]. Eventually, the quality of life of the majority of patients is restored [4,7]. However, studies indicate that a substantial minority do not regain their premorbid level of well-being and function [8,9]. Valid and reliable measures of health-related quality of life are necessary in order to describe the patients’ experiences of the diseases adequately and for intervention studies attempting to improve treatment efficacy. Therefore, there has been a growing interest within thyroidology in measuring patient-reported outcomes (PRO), leading to the development of a comprehensive PRO measuring thyroid-related quality of life, the ThyPRO. Due to the fact that individual thyroid diseases often co-exist (e.g., goiter and hyperthyroidism) and that treatment of one disease entity may lead to another (e.g., removal of a goiter leading to hypothyroidism), the ThyPRO was developed as a comprehensive thyroid-related measure, aimed at any benign thyroid disease.

The content of the ThyPRO addresses the impact of all benign thyroid diseases [10,11]. The validation of the current version has included evaluation of clinical validity in terms of known-groups comparisons and reliability in terms of internal consistency and test-retest reliability [12,13]. Further, the ThyPRO’s dimensionality or construct validity has been established by multi-trait scaling [12]. However, within such a framework, it is not possible to test the overall fit of a model [14], nor can misfit of items be modeled specifically.

The growing interest in applying the ThyPRO in clinical studies [7,15,16] and even in daily clinical practice has motivated efforts to develop shorter versions of the instrument as well as versions applicable to ecological momentary assessments. Development of such versions can be informed by the application of item response theory (IRT) models, which also provide a more detailed description of measurement precision and can provide data for interpretability of the ThyPRO. However, IRT models require additional, more detailed examinations of the dimensionality of the ThyPRO scales.

Structural equation models provide a latent variable modeling framework that is useful in detailed examinations of dimensionality. The measurement part of structural equation models can be used to assess the dimensionality of measured variables such as questionnaire items, using confirmatory factor analysis (CFA) for categorical data. Structural equation modeling can also test relationships among modeled latent variables (i.e., structural part of the models) [17-21]. We will exploit the former in the detailed analyses of the dimensionality of the ThyPRO scales, including overall test of model fit. We will use the structural part of the modeling approach when attempting to understand, through investigative modeling, any possible item misfit identified during the CFA step.

Thus, the purpose of the present study was to evaluate dimensionality of the ThyPRO scales in a sample of patients with a broad spectrum of thyroid diseases and to attempt to understand possible item misfit through investigative structural equation modeling.

## Methods

### The ThyPRO questionnaire

The current 85-item version of ThyPRO measures quality of life in 13 scales, covering physical (4 scales) and mental (2 scales) symptoms, function and well-being (3 scales) and participation/social function (4 scales) and one single item about overall quality of life. Content and scale structure were derived from a literature search [8] and from expert and patient interviews [10] and the development was conducted within a classical health-related quality of life theoretical framework [22-25]. Items are rated on a five-point scale from 0 = not at all to 4 = very much, with a reference period of 4 weeks. Thirteen scales are scored by reverting positively worded items and rescaling item scores from 0 (best QoL - absence of symptoms) to 100 (worst QoL – maximum level of symptoms) and taking the average across the items in the scale – i.e., standard summation and linear transformation.

### Patient population

The patient population comprised a cross-sectional sample of 907 patients attending two university hospital endocrine outpatient clinics during 2007 (Table 1 (For further details, see reference [13])). At one center, all consecutive patients newly referred to the clinic were invited to participate; at the other center, all patients attending the clinic during a specified period of time were invited, regardless of their referral time. Thus, patients from the former were mainly newly diagnosed whereas from the latter most were already receiving treatment. All common benign thyroid diagnoses were represented, as were various stages of disease and treatment. Clinical description of the patients included physical examination, ultrasonographic imaging and biochemical testing. The overall response rate was 69%. The project was approved by the local ethical committee (KF01 2006–1579) and the Danish Data Protection Agency and was registered at ClinicalTrials.gov (NCT00150033).

**Table 1:** **Characteristics of the N = 907 patients**

Women (%)/men	787 (87)/120
Age (mean (SD))	51 (15)
Diagnosis (n (%)):	
Diffuse non-toxic goitre	18 (2)
Multinodular non-toxic goitre	154 (17)
Uninodular non-toxic goitre	68 (7)
Solitary cyst	19 (2)
Multinodular toxic goitre	108 (12)
Uninodular toxic goitre	37 (4)
Graves’ hyperthyroidism	168 (19)
Graves’ orbitopathy	94 (10)
Autoimmune hypothyroidism	199 (22)
Subacute thyroiditis	9 (1)
Postpartum thyroiditis	8 (1)
Other thyroid disease	25 (3)
Months since diagnosis (median (range))*	27 (−0.9-607)
Thyroid treatment (n (%)):	
No thyroid treatment (ever)	283 (31)
Antithyroid medication	162 (18)
L-Thyroxine	292 (32)
Radioiodine	114 (13)
Thyroidectomy	132 (14)
Other treatment	4 (0.4)

*Negative durations reflect patient responding to the questionnaire before a final thyroid diagnosis was established.

### Statistical analyses

Prior to any of the statistical analyses mentioned below, a content analysis of each scale was performed to identify items which might be less associated with the remaining items in the same scale, and item pairs which might be closely related to one another after being accounted for by the scale (local item dependence). This was done to provide a content-based guidance to model fitting.

Then a one-factor confirmatory model for ordinal data was fitted to each individual scale [26,27], using Mplus (version 7.11) [28]. The ordinal items were regressed on the scale-factor by probit regressions estimated by a robust weighted least squares estimator with mean and variance adjustment (WLSMV) [28,29]. Appropriateness of the initial one-factor model for each scale was assessed by: 1) overall goodness-of-fit statistics including the comparative fit index (CFI) and the root mean square error of approximation (RMSEA), where CFI >0.95 and RMSEA < 0.08 were regarded as appropriate fit [30-34]; 2) magnitude of factor loadings; 3) model residual correlations (RC) and 4) modification indices (MI) [28,35]. For the latter three criteria, their magnitude was evaluated in comparison to other items in the scale and in an integrative manner, taking all three under consideration at once, so no strict thresholds were applied for each criterion. In general though, modification indices >100 and residual correlations > |.10| were taken as indices of lack of fit (local dependence or lack of convergent validity), but smaller values could also give rise to model revision considerations, if several indices pointed in the same direction; e.g., if an item had a modification index of 40 for a specific residual correlation (a “WITH”-statement in Mplus) and also had residual model correlations with several items. Revisions to improve model fit were based on both confirmatory factor modeling and content analysis, including specification of residual correlations among items, omission of poorly associated items from the models, and specification of sub-factors (for example among positively worded items in a scale). For scales where secondary factors seem plausible, a bifactor model was fitted to evaluate the dominance of the primary factor when secondary factors were modeled. A bifactor model specifies that each item is regressed on both a general and a group (secondary) factor, and the general and group factors are uncorrelated with each other [34,36-39]. The magnitude of loadings on the general and group factors were compared. The two-item scale on impaired sex life was not examined in this step, since a separate factor analysis of a two-item scale is not useful.

In an attempt to understand any possible item misfit identified through individual scale analyses, hypotheses which could explain the misfit were sought. These hypotheses were evaluated in a combined, investigational multidimensional model, where the individual scale factors were allowed to correlate freely. Also items were cross-loaded on multiple scale factors when necessary to explore a better understanding of item misfit. For example, if an item in a physical symptoms scale, e.g., “Palpitations”, had low own-factor loadings, it could be hypothesized that this was due to palpitations being influenced by mental health, e.g., as part of anxiety. Then cross-loading of this item on the mental symptoms scales would be specified and evaluated in the combined model.

In order to examine the stability of the model across various estimation techniques, the overall final model was compared with graded response multidimensional IRT models [40], fitted with the Mplus program [28]. For computational reasons, a 13-dimensional IRT model could not be estimated, so the model was broken down to four separate models, each containing scales with cross-loadings across scales. Stability was examined by comparing the estimated factor scores for each patient from the SEM vs. the IRT-model using intra-class correlations.

## Results

### Fitting unidimensional models to each individual ThyPRO scale

**Figure 1:**
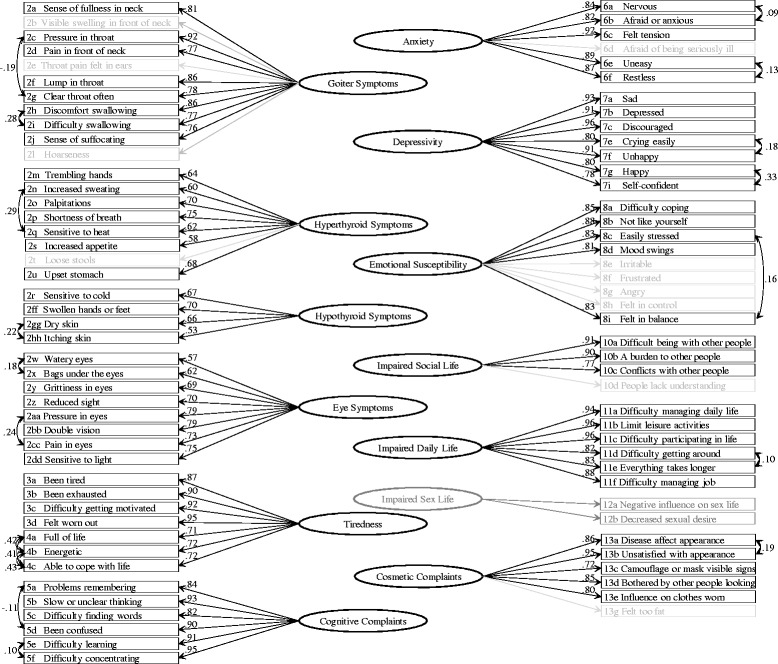
**Parameter estimates of the unidimensional confirmatory factor analyses of the revised ThyPRO scales.** Overall goodness-of-fit of the models are provided in the text. Grayed out items were omitted during model revision. The two-item Impaired Sexlife scale was not estimated.

Table 2 shows the results of the content analyses and the confirmatory factor analyses of the ThyPRO scales in their current version. In general, loadings were high in all scales and CFI was also high for the vast majority of scales. In contrast, for most scales, RMSEA was not below the 0.08 threshold for appropriate fit. Model parameters indicative of item misfit are presented to the right in Table 2. The consequential remodeling resulted in the revised scales presented in Figure 1 and the remodeling as well as the overall goodness-of-fit statistics are described separately for each scale in the following text.

**Table 2:** **Content analysis and confirmatory factor analyses of the individual ThyPRO scales**

**Scale and item**		**Possible misfit from content analysis**		**Initial unidimensional model** ^**a**^		
**Item #**	**Abbreviated item content**	**Unrelated content**	**Local dependence**	**Factor loading**	**Indication of local dependence** ^**b**^	**Indication of item misfit** ^**c**^
**Goiter Symptoms**				**CFI=0.95 RMSEA=0.16(0.15-0.16)**		
2a	Sense of fullness in neck			0.87	MI: LD with 2b	
2b	Visible swelling on neck			0.60	MI and RC: LD with 2a	Low loading
2c	Pressure in throat			0.90	RC: LD with 2g	
2d	Pain in front of neck		With 2e	0.71		
2e	Throat pain felt in ears	*	With 2d	0.60		Low loading and low IC
2f	Lump in throat			0.85		
2g	Clear throat often	*		0.69	MI: LD with 2l, RC: LD w. 2c	
2h	Discomfort swallowing		With 2i	0.94	MI: LD with 2i	
2i	Difficulty swallowing		With 2h	0.92	MI: LD with 2h	
2j	Sense of suffocating			0.73		
2l	Hoarseness	*		0.56	MI: LD with 2g	Low loading
**Hyperthyroid Symptoms**				**CFI=0.80 RMSEA=0.18(0.17-0.19)**		
2m	Trembling hands			0.60		
2n	Increased sweating		With 2o, 2p, 2q	0.71	MI: LD with 2q	
2o	Palpitations		With 2n, 2p	0.69		
2p	Shortness of breath		With 2n, 2o	0.64		
2q	Sensitive to heat		With 2n	0.70	MI: LD with 2n	
2s	Increased appetite			0.54		
2t	Loose stools		With 2u	0.75		Low IC and large neg. RCs
2u	Upset stomach		With 2t	0.80		
**Hypothyroid Symptoms**				**CFI=0.98 RMSEA=0.10(0.06-0.14)**		
2r	Sensitive to cold			0.56		
2ff	Swollen hands or feet			0.62		
2gg	Dry skin		With 2hh	0.86	RC: LD with 2hh	
2hh	Itching skin		With 2gg	0.63	RC: LD with 2gg	
**Eye Symptoms**				**CFI=0.94 RMSEA=0.11(0.09-0.11)**		
2w	Watery eyes		With 2y, cc, dd	0.62	MI and RC: LD with 2x	
2x	Bags under the eyes			0.59	MI and RC: LD with 2w	
2y	Grittiness in eyes		With 2w, 2cc, 2dd	0.74		
2z	Reduced sight	*		0.68		
2aa	Pressure in eyes		With 2cc	0.87	MI: LD with 2cc	
2bb	Double vision	*		0.70		
2cc	Pain in eyes		With 2w, y, dd, aa	0.86	MI: LD with 2aa	
2dd	Sensitive to light		With 2w, y, cc	0.70		
**Tiredness**				**CFI=0.99 RMSEA=0.28(0.26-0.28)**		
3a	Been tired			0.90	MI: LD with 3b	
3b	Been exhausted			0.93	MI: LD with 3a	
3c	Difficult get motivated			0.89		
3d	Felt worn out			0.91		
4a	Full of life		With 4b, 4c	0.93	MI and RC: LD with 4b, 4c	
4b	Energetic		With 4a, 4c	0.98	MI and RC: LD with 4a, 4c	
4c	Able to cope with life		With 4a, 4b	0.95	MI and RC: LD with 4a, 4b	
**Cognitive Complaints**				**CFI=0.99 RMSEA=0.13(0.11-0.15)**		
5a	Problems remembering		With 5c	0.87	RC: LD with 5d	
5b	Slow or unclear thinking		With 5f	0.94		
5c	Difficulty finding words		With 5a	0.85		
5d	Been confused	*		0.85	RC: LD with 5a	
5e	Difficulty learning			0.92	MI: LD with 5f	
5f	Difficulty concentrating		With 5b	0.91	MI: LD with 5e	
**Anxiety**				**CFI=0.97 RMSEA=0.16(0.14-0.18)**		
6a	Nervous			0.90	MI: LD with 6b	
6b	Afraid or anxious			0.90	MI: LD with 6a	
6c	Felt tension			0.88		
6d	Afraid being seriously ill	*		0.70		Low loading, neg. RC’s
6e	Uneasy		With 6f	0.92	MI: LD with 6f	
6f	Restless		With 6e	0.80	MI: LD with 6e	
**Depressivity**				**CFI=0.96 RMSEA=0.24(0.23-0.26)**		
7a	Sad			0.95		
7b	Depressed		With 7c	0.92		
7c	Discouraged		With 7b	0.94		
7e	Crying easily	*		0.79	MI: LD with 7f	
7f	Unhappy		With 7g	0.92	MI: LD with 7e	
7g	Happy		With 7i, 7f	0.76	MI: LD with 7i	
7i	Self-confident	*	With 7g	0.74	MI: LD with 7g	
**Emotional Susceptibility**				**CFI=0.92 RMSEA=0.24(0.23-0.25)**		
8a	Difficulty coping			0.80		
8b	Not like yourself			0.80		
8c	Easily stressed			0.81	MI: LD with 8i	
8d	Mood swings			0.88		
8e	Irritable		With 8g	0.89		Large neg. RC
8f	Frustrated			0.91		MI: LD with many other items
8g	Angry		With 8e	0.80		MI: LD with many other items
8h	Felt in control		With 8i	0.87		MI: LD with many, large neg. RC’s
8i	Felt in balance		With 8h	0.91	MI: LD with 8i, 8c	
**Impaired Social Life**				**CFI=0.99 RMSEA=0.08(0.05-0.13)**		
10a	Difficult with people			0.90		
10b	A burden to people			0.89		
10c	Conflicts with people			0.80		
10e	Others lack understanding	*		0.71		Low loading, neg. RC’s
**Impaired Daily Life**				**CFI=0.99 RMSEA=0.10(0.08-0.12)**		
11a	Difficult manage life			0.94		
11b	Limit leisure activities		With 11f	0.95	MI: LD with 11f	
11c	Difficult participate in life			0.96		
11d	Difficult getting around	*		0.84	MI: LD with 11e	
11e	Everything takes longer	*		0.85	MI: LD with 11d	
11f	Difficulty managing job		With 11b	0.88	MI: LD with 11b	
**Cosmetic Concern**				**CFI=0.98 RMSEA=0.10(0.08-0.12)**		
13a	Disease affect appearance		With 13b	0.83	MI: LD with 13b	
13b	Unsatisfied appearance		With 13a	0.98	MI: LD with 13a	
13c	Camouflage visible signs			0.79		
13d	Other people looking			0.83		
13e	Influence on clothes worn			0.79		
13g	Felt too fat	*		0.65		Low loading

^a^CFI: Comparative fit index, RMSEA: Root mean square error of approximation with 90% confidence interval.

^b^MI: Modification indices, LD: local dependence, RC: model residual correlation.

^c^IC: Model inter-item correlation.

Left part of the table presents the results of the initial content analyses. The results of the initial unidimensional confirmatory factor analyses are presented in the right part of the table: overall goodness-of-fit, factor loadings as well as the indices of possible local dependency and item misfit which lead to remodeling in next steps of the analyses.

#### Goiter Symptoms

Three items were problematic (2b Visible swelling in front of neck, 2e Throat pain felt in ears and 2l Hoarseness), with relatively low loadings and indication of local dependence with other items. Two of these items were identified prior to the modeling as potentially less related to the concept. Two instances of local dependence among other items were identified (2c Pressure in throat vs. 2 g Need to clear throat often and 2 h Discomfort swallowing vs. 2i Difficulty swallowing, Table 2). When omitting the three items and modeling the local dependencies, an appropriately fitting unidimensional model was reached (Figure 1, CFI = 0.99, RMSEA(90%CI) = 0.08(0.07-0.09)).

#### Hyperthyroid Symptoms

For one pair of items (2n Increased sweating vs. 2q Sensitive to heat), the modification index suggested local dependence and one item (2t Loose stools) had large negative residual correlations with other items, when the initial model was estimated. When omitting the latter and fitting the local dependence, a unidimensional model obtained an appropriate fit to the data (Figure 1, CFI = 0.97 RMSEA(90%CI) = 0.06(0.05-0.08)).

#### Hypothyroid Symptoms

When modeling the expected local dependence between the items concerning skin (2gg Dry skin vs. 2hh Itching skin), an appropriate fit between an overall unidimensional model and data was demonstrated for this scale (Figure 1, CFI = 1.0 RMSEA(90%CI) = 0.00(0.00-0.09).

#### Eye Symptoms

With the specification of two local dependence-pairs (2w Watery eyes vs. 2x Bags under eyes and 2aa Pressure in eyes vs. 2cc Pain in eyes), an appropriate fit of a unidimensional model was found (Figure 1, CFI = 0.99 RMSEA(90%CI) = 0.06(0.04-0.07).

#### Tiredness

**Figure 2:**
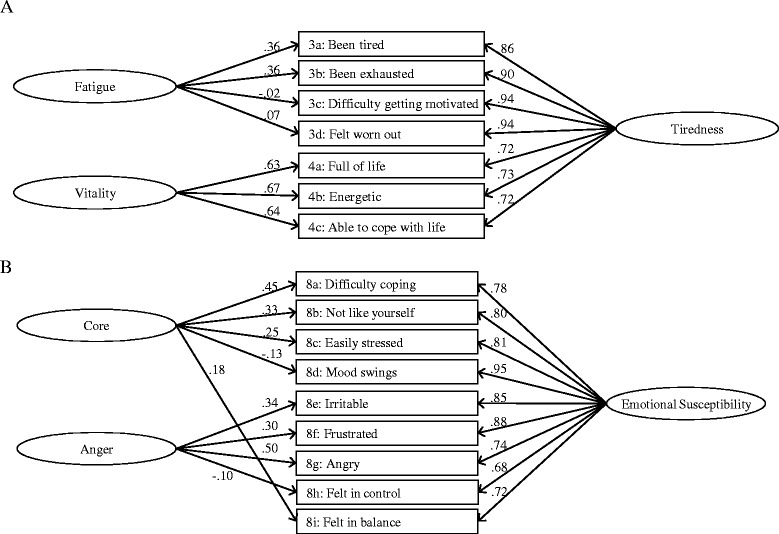
**Bi-factor models for the Tiredness (Panel A) and the Emotional Susceptibility (Panel B) scales.**

Despite quite high factor loadings, overall goodness-of-fit was poor for this scale. To avoid floor problems, three items had been formulated positively for this scale. The positively worded items had high positive residual correlations and modification indices. A bi-factor model distinguishing positively from negatively worded items was therefore evaluated (Figure 2, Panel A). Although the positively worded items had high loadings on the positive factor (Vitality), loadings on the general factor were higher. When modeling the local dependence among positively worded items as residual correlations and also allowing for the local dependence between 3a and 3b, the model had good fit (Figure 1, CFI = 1.0, RMSEA(90%CI) = 0.02 (0.00-0.04).

#### Cognitive Complaints

All items had high loadings in the initial model (Table 2). When specifying two pairs of local dependence, suggested by modification indices (5a Problems remembering vs. 5d Been confused and 5e Difficulty learning vs. 5f Difficulty concentrating), overall model fit was appropriate (Figure 1, CFI = 1.0 RMSEA(90%CI) = 0.07(0.05-0.09)).

#### Anxiety

According to overall goodness-of-fit indices, the initial model did not obtain an appropriate fit to the data (Table 2). When fitting a model by excluding the item identified as less related with the other items (6d Afraid being seriously ill) and by specifying two item pairs with local dependence (6a Nervous vs. 6b Afraid or anxious and 6e Uneasy and 6f Restless), appropriate fit was obtained (Figure 1, CFI = 1.0, RMSEA(90%CI) = 0.07(0.04-0.10)).

#### Depressivity

All items had high loadings (Table 2). However, only after specification of two local dependence pairs (7e Crying easily vs. 7f Unhappy and 7 g Happy vs. 7i Self-confident), was an appropriate overall fit to data reached (Figure 1, CFI = 1.0 RMSEA(90%CI) = 0.07 (0.05-0.09)).

#### Emotional Susceptibility

In contrast to most other concepts measured by ThyPRO, this scale measures a unique aspect of mental health identified through qualitative analysis of patient interviews. Thus, it is not classically described as a separate concept. It is, however, an important aspect according to the patients and a prominent feature particularly among patients with thyroid autoimmunity [10]. According to the overall fit indices, these items do not appropriately conform to a unidimensional model, despite high factor loadings (Table 2). Several items had high inter-item residual correlations and were attempted to be modeled as a separate “Anger” sub-factor (Figure 2, Panel B). However, as shown in Figure 2, the sub-factor loadings were rather low. Four items had to be omitted in order to obtain appropriate fit between a unidimensional model and the data (Figure 1, CFI = 1.0 RMSEA(90%CI) = 0.08(0.05-0.11)). A local dependence (8c Easily stressed vs. 8i Felt in balance) was also modeled.

#### Impaired Social Life

Appropriate, albeit not good overall goodness-of-fit indices were found for the initial unidimensional model. Excluding the lowest-loading item (10d People lack understanding), which was also pre-specified as possibly less associated, resulted in a just-identified model, hence with perfect fit (Figure 1, CFI = 1.0 RMSEA(90%CI) = 0.00(0.00-0.00)).

#### Impaired Daily Life

With the specification of one local dependence (11d Difficulty getting around vs. 11e Everything takes longer), a unidimensional model fit the data appropriately (Figure 1, CFI = 1.0, RMSEA (90%CI) = 0.08(0.07-0.10)).

#### Cosmetic Complaints

The initial unidimensional model had almost appropriate goodness-of-fit indices (Table 2). When modeling one local dependence (13a Disease affect appearance vs. 13b Unsatisfied with appearance) and leaving out the very nonspecific item concerning feeling too fat (13g), a good fit between model and data was found (Figure 1, CFI = 1.0 RMSEA(90%CI) = 0.05(0.02-0.08)).

### Investigative modeling of possible item misfit within one combined multidimensional model

This investigative model is presented in Table 3. The hypotheses concerning the reason for misfit of the omitted items are presented in the second column of the table. In these models, the possible sub-factors tested in bifactor models (Figure 2) were specified as residual correlations among the involved items. In the third column of Table 3, it is specified how these hypotheses were modeled in the combined multidimensional model, where all the factors were evaluated simultaneously and were allowed to correlate freely. The results of this investigative modeling are described in the rightmost column of Table 3. Generally, a closer association was found between items and their own scale for the items in the multidimensional model (e.g. items 2e, 2 t and 10e), than in the unidimensional model for each scale. For most items, the hypothesized explanations for the apparent misfit were confirmed. Thus, 2b Visible swelling on neck was indeed associated with Cosmetic Complaints (−0.23). Item 2l Hoarseness did load also on the Hypothyroid Symptoms scale (0.22), 2t Loose stools was negatively associated with particularly Hypothyroid Symptoms (−0.55), and a negative association between 6d Afraid of being seriously ill and time since diagnosis was found. In contrast, no relationship between item 10e Other people lack understanding and mental health scales was found. Item13g Feeling too fat was associated with both Hypothyroid Symptoms (−0.16), Anxiety (−0.22) and Depressivity (0.15), and had low loading on its own factor (0.53).

**Table 3:** **For each item which was omitted during the single-scale analyses, hypotheses regarding possible reasons for misfit were formulated, modeled and tested as specified**

**Item**	**Hypothesized reason for misfit**	**Investigative modeling of the hypothesized reason for misfit**	**Results of the investigative modeling**
2b Visible swelling on neck from the Goiter Symptoms scale	May relate to cosmetic concerns, rather than being a symptom	Item was allowed to cross-load on the Cosmetic Complaints factor	Loaded −0.23 on the Cosmetic Complaints factor.
			Loading on own factor: 0.68
2e Throat pain felt in ears from the Goiter Symptoms scale	May be relevant only for patients with subacute thyroiditis, during the acute inflammatory phase.	No marker of acute inflammation is available in the clinical database describing the patients. Only 9 patients in this sample had subacute thyroiditis	Extraneous modeling not possible.
			Loading on own factor in the full model: 0.75
2l Hoarseness from the Goiter Symptoms scale	Hoarseness is also a classical symptom of hypothyroidism. Might relate more to hypothyroidism than to goiter.	Item was allowed to cross-load on the Hypothyroid Symptoms factor	Loaded 0.22 on Hypothyroid Symptoms factor.
			Loading on own factor: 0.46
2t Loose stools from the Hyperthyroid Symptoms scale	Might be a non-specific physical symptom	Item was allowed to load on the other physical symptoms factors, except for Eye Symptoms	Loaded −0.15 on Goiter Symptoms factor and −0.55 on Hypothyroid Symptoms.
			Loading on own factor: 1.20
6d Afraid of being seriously ill from the Anxiety scale	May be related to not being fully examined yet, and thus an initial fear of e.g. cancer has not yet been ruled out completely	Item was regressed on time since diagnosis.	A significant negative association with time since diagnosis was found
10e Other people lack understanding from the Impaired Social Life scale	May relate more to depressive mood and emotional distress than the other items in the Social Life scale	Item was allowed to cross-load on the Depressivity and the Emotional Susceptibility factor	No significant loading on Depressivity or Emotional Susceptibility was found.
			Loading on own factor: 1.08
13g Felt too fat from the Cosmetic Complaints scale	Weight gain is often experienced during hypothyroidism. Feeling too fat may also relate more to a negative self-esteem aspect of depressive mood	Item was allowed to cross-load on the Hypothyroid Symptoms and Depressivity and Anxiety factors	Loaded −0.16 on Hypothyroid Symptoms factor, −0.22 on Anxiety and 0.15 on Depressivity factor.
			Loading on own factor: 0.53

In analyses of concordance of results from SEM and the IRT-model, high intra-class correlation coefficients (0.94-0.99) were found for all 13 scales, when comparing factor scores derived by the SEM with IRT score estimates (Table 4).

**Table 4:** **Comparison of individual factor-scores derived from the ordinal confirmatory factor analysis approach with the factor scores derived from the item response theory (IRT) approach**

	**Ordinal vs. IRT factor scores intra-class correlation coefficients**
Goiter Symptoms	0.99
Hyperthyroid Symptoms	0.98
Hypothyroid Symptoms	0.94
Eye Symptoms	0.96
Tiredness	0.98
Cognitive Complaints	0.98
Anxiety	0.97
Depressivity	0.98
Emotional Susceptibility	0.98
Impaired Social Life	0.95
Impaired Daily Life	0.94
Impaired Sex Life	0.95
Cosmetic Complaints	0.98

## Discussion

The purpose of the present study was to evaluate the dimensionality of the ThyPRO scales and to detect and understand potential item misfit. Since an established scale structure already exists for the ThyPRO, we used a combination of confirmatory factor analyses of the individual scales and a combined multidimensional model comprising all 13 ThyPRO scales. In case of misfit for each individual scale, we revised the model to achieve the best description of data.

In general, items had high loadings on their own factors and the comparative fit indices were high, but for the majority of the scales, the root means square error of approximation indicated that a simple unidimensional model was not fitting the data sufficiently well. Based on prior expectations informed by content analyses, modeling results (model inter-item correlations and model residual correlations) and on model modification indices, the models were adjusted in order to reduce the overall misfit. For all scales, an appropriate fit according to the overall goodness-of-fit indices could be reached. During this process, a total of 11 items were left out of the models and 18 residual correlations indicating local dependence were specified.

In most instances, the magnitude of the residual correlations representing local dependencies was small, and the loading on the relevant general factor was still high. Most of the residual correlations were among very similarly worded items. Such local dependencies are not problematic for the current scoring of the ThyPRO, but may lead researchers to overestimate the precision gained by the instrument, because locally dependent items provide less measurement precision than assumed by standard psychometric analyses [41]. Moreover, one of the items involved in such pairs would be potential candidates for omission in future IRT-modeling of the instrument and in the development of abbreviated versions of the ThyPRO.

However, such item reduction should be done with caution and should take clinical analyses and considerations into account.

Although positively worded items did tend to exhibit residual correlations, we found no consistent evidence of a method factor among the positively worded items. Similar studies with other outcome measures have previously found substantial influence of the value of the wording [36,42-44], whereas other studies either did not identify such an effect [45] or the identified effect had only minor influence on the results regarding the substantive factor [46].

We attempted to model potential item misfit identified during the dimensionality analyses of the existing ThyPRO scales. This was done within a model including all scales, which were allowed to correlate, in order to allow for cross-loadings of items to be examined and in order to evaluate if possible misfit identified during individual scale analyses was due to interrelation with other factors. In doing so, the hypothesized reason for misfit was confirmed in five of seven items: Item 2b, about visibility of the goiter, cross-loaded on Cosmetic Complaints. Item 2t, Loose stools, had a large negative loading on Hypothyroid Symptoms, as had 2l, Hoarseness. Both constipation and hoarseness are indeed salient and classical features of hypothyroidism [47]. The rather non-specific item 13g, Feeling too fat, which is a common complaint among hypothyroid patients and among hyperthyroid patients after treatment, had cross-loadings on several other scales and low loading on its own factor, also when modeled multidimensionally. Thus, these four items are very strong candidates for item reduction when developing abbreviated and focused versions of the scales or when fitting models where unidimensionality is a strong assumption, for example as in unidimensional IRT models.

A unique “duration of disease”-effect was observed for one item. Item 6d, Afraid of being seriously ill was negatively associated with time since diagnosis, indicating that the responses to this item reflects a relevant concern early in the disease course, for instance of a goiter being malignant, a concern that wanes as the diagnosis becomes more firmly established and malignancy thus ruled out. It thus measures something different from the other items in the scale, which are more classical indicators of an anxious state.

As an analysis of the robustness and appropriateness of the ordinal confirmatory WLSMV factor analysis, an alternative multidimensional IRT-based analysis was performed. Individual factor scores derived from each of these approaches were very similar, as illustrated by very high intra-class correlation coefficients. This corroborates the current simple scoring approach and the results of the present analyses.

The use of theoretically driven analyses within a clinically well-described and relatively (for thyroid diseases) large sample was a strength of this study. However, the analyses were carried out in one sample and should ideally be confirmed in a new independent sample. Furthermore, although the present sample comprised patients in all stages of disease and treatment, stability of the factor structure across time could not be evaluated, since the data did not contain longitudinal measurements.

In conclusion, each of the ThyPRO scales could be appropriately represented by a unidimensional model after minor revisions. Eleven items were identified in the unidimensional models as potentially misfitting and understood further by multidimensional modeling. Thus, overall the previous initial examinations of the construct validity of the scales [12] were corroborated using a more elaborate technique. Further, advanced psychometric modeling such as IRT, with strong assumptions about dimensionality, can be applied to the reduced scales. Finally, the locally dependent items identified here are strong candidates for removal, in future item reduction processes.
